# Comparative genomic profiling of Dutch clinical *Bordetella pertussis *isolates using DNA microarrays: Identification of genes absent from epidemic strains

**DOI:** 10.1186/1471-2164-9-311

**Published:** 2008-06-30

**Authors:** Audrey J King, Tamara van Gorkom, Jeroen LA Pennings, Han GJ van der Heide, Qiushui He, Dimitri Diavatopoulos, Kees Heuvelman, Marjolein van Gent, Karin van Leeuwen, Frits R Mooi

**Affiliations:** 1 Laboratory for Infectious Diseases and Screening (LIS) Centre for Infectious Disease Control, National Institute for Public Health and the Environment (RIVM), Bilthoven, The Netherlands; 2 Laboratory for Health Protection Research, National Institute for Public Health and the Environment (RIVM), Bilthoven, The Netherlands; 3 Pertussis Reference Laboratory, National Public Health Institute, Turku, Finland; 4 Department of Microbiology and Immunology, University of Melbourne, Victoria, Australia

## Abstract

**Background:**

Whooping cough caused by *Bordetella pertussis *in humans, is re-emerging in many countries despite vaccination. Several studies have shown that significant shifts have occurred in the *B. pertussis *population resulting in antigenic divergence between vaccine strains and circulating strains and suggesting pathogen adaptation. In the Netherlands, the resurgence of pertussis is associated with the rise of *B. pertussis *strains with an altered promoter region for pertussis toxin (*ptxP3*).

**Results:**

We used Multi-Locus Sequence Typing (MLST), Multiple-Locus Variable Number of Tandem Repeat Analysis (MLVA) and microarray-based comparative genomic hybridization (CGH) to characterize the *ptxP3 *strains associated with the Dutch epidemic. For CGH analysis, we developed an oligonucleotide (70-mers) microarray consisting of 3,581 oligonucleotides representing 94% of the gene repertoire of the *B. pertussis *strain Tohama I. Nine different MLST profiles and 38 different MLVA types were found in the period 1993 to 2004. Forty-three Dutch clinical isolates were analyzed with CGH, 98 genes were found to be absent in at least one of the *B. pertussis *strains tested, these genes were clustered in 8 distinct regions of difference.

**Conclusion:**

The presented MLST, MLVA and CGH-analysis identified distinctive characteristics of *ptxP3 B. pertussis *strains -the most prominent of which was a genomic deletion removing about 23,000 bp. We propose a model for the emergence of *ptxP3 *strains.

## Background

*Bordetella bronchiseptica, Bordetella parapertussis *and *Bordetella pertussis *are closely related respiratory pathogens that infect mammalian species.*B. bronchiseptica *causes chronic respiratory tract infections in a wide variety of mammals but has only been sporadically isolated from humans [1,2]. *B. parapertussis *consists of two distinct lineages that infect sheep and humans, respectively [3].* B. pertussis*is is a human pathogen that causes pertussis, also known as whooping cough, a disease that is particularly severe in infants.

For about 50 years, many countries have been immunizing young children with pertussis vaccines to control disease. Despite vaccination, pertussis remains endemic, with regular epidemic outbreaks. World-wide, whooping cough causes up to 300,000 deaths every year, mostly among unvaccinated infants [4]. Even in countries with a high vaccination coverage, a significant increase in the pertussis incidence has been observed since the 1990s [5-7]. In the Netherlands, such an increase in pertussis has been observed since 1996 [8].

The resurgence of pertussis in countries with high vaccination coverage has been attributed to a number of factors, including increased awareness with regard to the disease, improved diagnostics, waning vaccine-induced immunity and pathogen adaptation [4,7]. Consistent with pathogen adaptation, numerous studies have demonstrated that the *B. pertussis *population has changed in several countries where vaccination has been implemented since the 1950s [9-13]. In particular, antigenic divergence was found between circulating strains and vaccine strains with respect to pertussis toxin (Ptx) and pertactin (Prn).

In the Netherlands this divergence between vaccine and circulating strains has played a role in the reemergence of pertussis [7,14]. More recent studies have also found polymorphism in other surface proteins, including tracheal colonization factor A (TcfA) [15,16], the serotype 2 and 3 fimbrial subunits (Fim2 and Fim3) [15,17-19] and in the promoter region for the *ptx *operon [20]. The latter mutation was found in the Netherlands since the 1990s and we observed that strains with a particular allele of the *ptx *promoter (*ptxP*), i.e. *ptxP3*, have expanded in the Dutch *B. pertussis *population. The increase in frequency of *ptxP3 *strains coincided with the increase in pertussis notifications in the Netherlands. Moreover *B. pertussis *strains carrying this novel allele for the pertussis toxin promoter were shown to confer increased virulence [21].

In recent years genomic tools like DNA microarrays have been used for comparison of the genetic composition of different strains within a species. Several microarray-platforms have been developed and applied to specifically address questions related to the genome of *B. pertussis *[22-25].

In the study presented here we used Multi-Locus Sequence Typing (MLST), Multiple-Locus Variable Number of Tandem repeat Analysis (MLVA) and a microarray-based Comparative Genomic Hybridization (CGH) method to investigate the heterogeneity of the strains dominating the current Dutch pertussis epidemics, in particularly the *ptxP3 *strains. We have developed an oligonucleotide microarray representing 94% of the gene repertoire of the *B. pertussis *Tohama I strain, of which the genome has been sequenced [26]. This microarray allowed us to study the gene contents of the strains involved in the Dutch epidemics and thus to identify (other) polymorphic loci associated with epidemic phenotype. Representative Dutch isolates from 1993 to 2004 were selected since pertussis epidemics have been occurring regularly in the Netherlands since 1996. We aimed to identify MLST, MLVA and gene profiles associated with the Dutch epidemics and to determine the characteristics and heterogeneity of those epidemic strains.

## Results

Dutch strains were selected from the period 1993–2004. The period 1993–1995 was characterized by a relative low level of notifications. In 1996 a sudden increase in notifications was observed and this high level has been maintained until today [5].

### MLST analyses

Three genes known to be polymorphic in the period 1993–2004, *ptxP*, *fim3 *and *prn*, were selected for the MLST analysis of 158 strains. The *ptxP *gene occurred as two alleles, *ptxP1 *and *ptxP3*. The *fim3 *gene occurred as four alleles (*fim3-1, fim3-2, fim3-3, fim3-4*), while three *prn *alleles were observed (*prn1*,*prn2 *and *prn3*).

**Figure 1:**
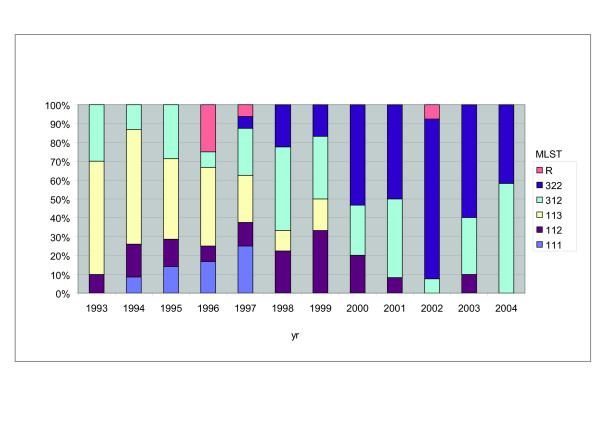
**Temporal trends in MLST frequencies of Dutch *B. pertussis *strains from 1993 to 2004**. The MLST designation was based on the allele number in the order *ptxP*, *fim3 *and *prn*. E.g. MLST113 represents strains with *ptxP1*, *fim3-1 and prn3*. MLSTs found in frequencies less then 1.5% in the whole period were combined into a single group designated R.

We observed 9 different MLST-profiles in the period from 1993 to 2004 (Fig. 1). A number of MLSTs (i.e. MLST141, MLST311, MLST313 and MLST323) were found in low frequencies in the whole period (< 1.5%) and combined into a single group designated R. The highest frequency of the minor MLSTs (25%) was found in 1996. For clarity, we will discuss first discuss MLSTs with *ptxP1*, followed by those harboring *ptxP3*. Strains with the *ptxP1 *allele were gradually replaced by *ptxP3 *strains, which were predominant from 1998 on. The *ptxP1 *allele was linked to *fim3-1 *(linkage 100%) and three *prn *alleles, *prn1*, *prn2 *and *prn3 *(linkage, respectively, 16%, 30% and 54%). MLST113 predominated until 1996 (frequencies 61% to 42%) and showed a gradual decrease in frequency from 1996 to 1999, after which it was not detected. MLST111 was detected in the period 1994–1997 (frequencies 9%–25%). MLST112 strains were found in the whole period, with the exception of the years 2002 and 2004, in frequencies of 8%–22%.

In contrast to *ptxP1*, *ptxP3 *was linked to two *fim3 *alleles, *fim3-1 *and *fim3-2 *(linkage, respectively, 52% and 48%). As was observed for *ptxP1*, *ptxP3 *was associated with *prn1*, *prn2 *and *prn3 *(linkage 2%, 96%, 2%). Thus in contrast to *ptxP1*, *ptxP3 *was mostly linked to *prn2*. Two predominant MLSTs associated with *ptxP3 *were observed, MLST312 and MLST322. MLST312 was found throughout the whole period and predominated in 1998 and 2004 (frequencies 44% and 58%, respectively). MLST322 was first detected in 1997 (frequency 6%) and predominated in the period 2000 to 2003 (frequencies 50% to 85%).

### MLVA analyses

**Figure 2:**
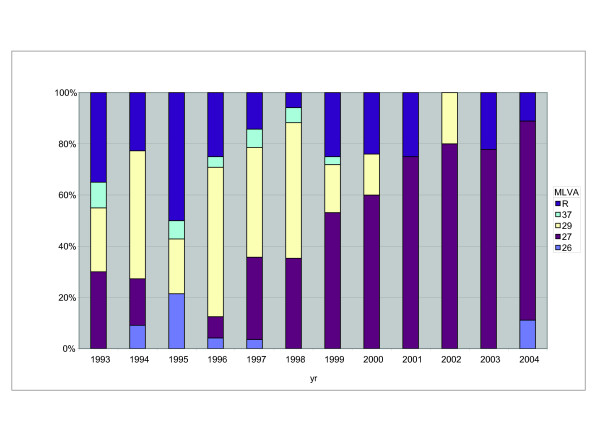
**Temporal trends in MLVA frequencies of Dutch *B. pertussis *strains from 1993 to 2004**. Frequencies of different MLVA-types are shown. MLVA types found in frequencies lower than 1.6% in the whole period were combined into a single group designated R.

A collection of 222 clinical isolates from the period 1993 to 2004 were typed by MLVA and 38 types were found. Of the 38 MLVA types, 6 have not been described before (see Additional file 1). Most types (64%) were found in a single isolate only and these were combined into a single group, designated R (Fig. 2). The remaining four types MLVA26, MLVA27, MLVA29 and MLVA37 were found in frequencies of, respectively, 4%, 41%, 30% and 4% in the whole period. In the period 1993–2004, MLVA29 was found in frequencies of 16%–58%, but was not observed in 2001, 2003 and 2004 (Fig. 2). It was the predominant type in the year 1996 (frequency 58%). MLVA27 was not detected in 1995 and was observed in frequencies of 18% to 78% in the other years. It was the predominant type from 1999 to 2004. Compared to MLVA27 and MLVA29, MLVA26 and MLVA37 were detected in much lower frequencies (3%–21%), and only in the years 1993–1999. In 1995 the highest frequency of minor types was observed (50%).

**Figure 3:**
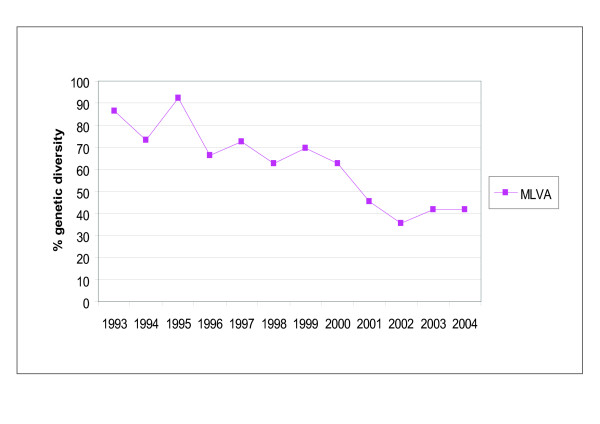
**Genetic diversity of the Dutch *B. pertussis *population in the period 1993 to 2004**. The genetic diversity was calculated for each year using MLVA frequencies.

The genetic diversity based on MLVA types showed a decrease from approximately 80% in the early nineties to 42% in 2003 and 2004 (Fig. 3 and see Additional file 2).

**Figure 4:**
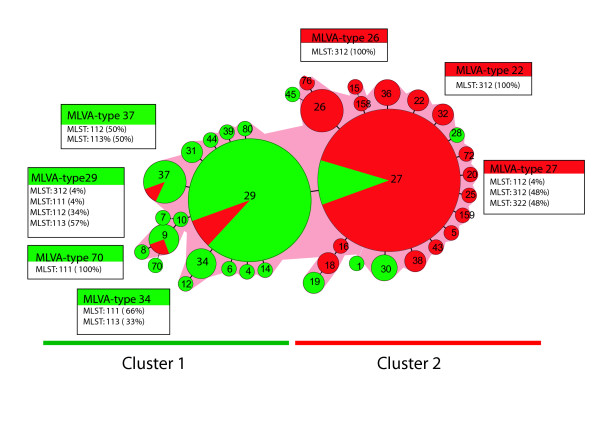
**Minimum spanning tree based on MLVA of Dutch *B. pertussis *strains isolated from 1993 to 2004**. Each circle represents a different MLVA-type, indicated by the number in the middle of the circle. The size of the circle is related to the number of isolates within the particular MLVA-type. Green and red colors indicate, respectively the *ptxP1 and ptxP3 *allele frequencies within MLVA. The distribution of the MLST types is shown in boxes for the most commonly found MLVA types. In some cases the MLST types are not 100% due to the fact that not all strains were types by MLST.

The relationship between MLST and MLVA types was investigated by constructing a minimum spanning tree based on MLVA-types of 222 *B. pertussis *strains (Fig. 4). Two main clusters were observed. The majority of the strains in cluster 1 belonged to the related MLVA types 29 and 37. Most of the strains (94.7%) of MLVA type 29 and 37 had the *ptxP1 *allele (MLST-profiles 111, 112 or 113). Similarly, most strains in cluster 2 belonged to the related MLVA types 27 and 26 and most (92%) of the strains of these MLVA types carried *ptxP3 *allele (MLST-profiles 312 or 322).

### CGH analyses

**Figure 5:**
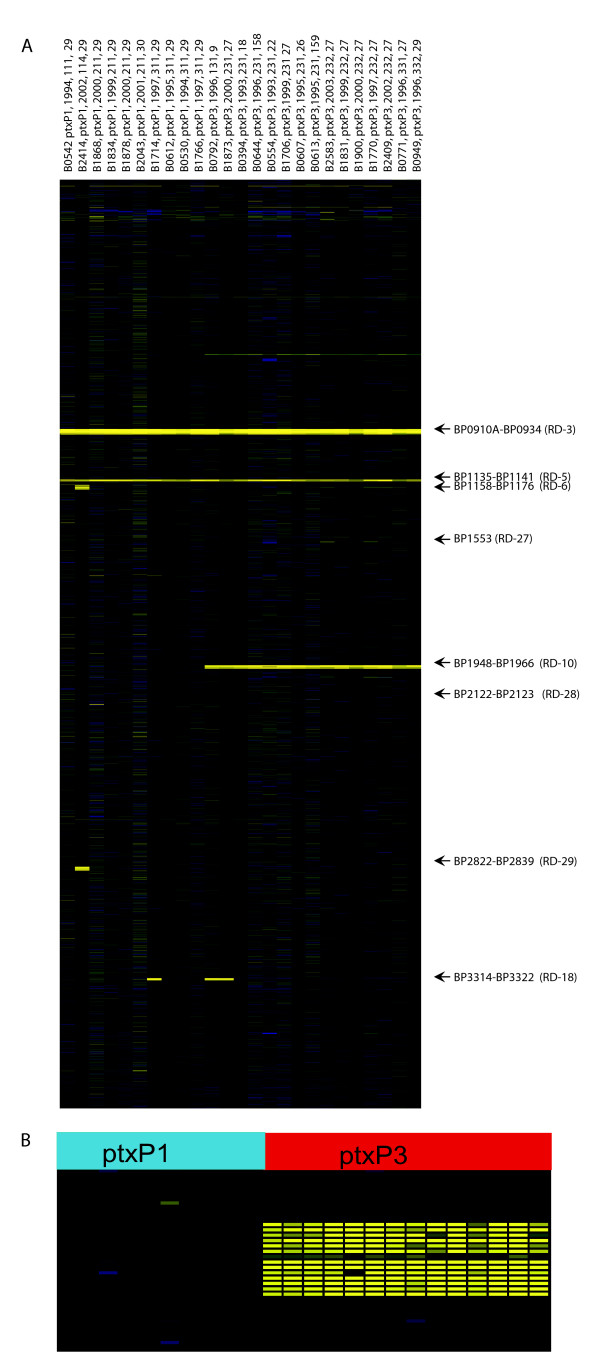
**CGH data for Dutch clinical isolates**. CGH analysis of 25 different *B. pertussis *isolates. **A**. Each column represents one strain. Strain designation, *ptxP *type, year of isolation, MLST type and MLVA type are indicated above the column. Each row represents one ORF (in *B. pertussis *Tohama I gene order). Text on the right indicates prominent RD's. **B**. Gene content of the locus containing BP1948-BP1966 genes (RD-10) as determined by CGH. Each column represents one strain, strains are sorted by the *ptxP *type in order to visualize the difference found in this region between *ptxP1 *and *ptxP3 *type strains and each row represents one ORF (in *B. pertussis *Tohama I gene order).

Microarray-based comparative genomic hybridization (CGH) with the *B. pertussis *oligonucleotide microarray based on the Tohama I genome was used for genomic analyses of the *ptxP1 *and *ptxP3 *lineages in order to determine if they differed with respect to gene content. Forty-three Dutch clinical isolates, isolated in the period 1993–2004, were analyzed with CGH, seventeen *ptxP1 *strains and twenty-six *ptxP3 *strains. Complete hybridization profiles for twenty-five *B. pertussis *isolates are presented in Fig. 5. Of the 3,581 genes spotted on the microarray, 98 (2.7%) were found to be absent in at least one of the *B. pertussis *strains tested. The 98 genes were clustered in eight contiguous loci or regions of difference (RDs) (Table 1).

**Table 1:** Presence of regions of difference in Dutch clinical isolates from 1993 to 2004

**Region of difference Locus:**	**BP-number**	**No. of genes**	**Size (kb)**	***ptxP1 *lineage**	***ptxP3 *lineage**
*RD-3*	BP0910A-BP0937	23	24.9	0% (nr strains)	0% (nr strains)
*RD-5*	BP1135-BP1141	7	8.6	0% (nr strains)	0% (nr strains)
*RD-6*	BP1158-BP1176	19	18.7	94% (nr strains)	100% (nr strains)
*RD-27*	BP1553	1	0.8	82% (nr strains)	100% (nr strains)
*RD-10*	BP1948-BP1966	18	22.7	100% (nr strains)	0% (nr strains)
*RD-28*	BP2122-BP2123	2	1.7	94% (nr strains)	100% (nr strains)
*RD-29*	BP2822-BP2839	17	16.9	94% (nr strains)	100% (nr strains)
*RD-18*	BP3314-BP3322	9	9.4	94% (nr strains)	92% (nr strains)
RD-1*	BP0515-BP0516	2			

Regions of difference (RDs) are numbered according to Brinig *et al *[23]. RD-1* is part of RD-1 as was found by Brinig *et al *[23]. The percentage of strains harbouring the region and the number of strains analyzed are indicated. Size, refers to the size of the deletion.

All strains isolated between 1993 and 2004 contained a deletion in two RDs relative to the Tohama I strain: RD-3 (23 genes, 24.9 kb) and RD-5(7 genes, 8.6 kb). The 22 gene products of RD-3 (see Additional file 3) were classified in gene class groups (Table 2). Four (17%) cell envelope proteins, three (13%) regulators and 2 (9%) proteins involved in transport and binding were observed. Five (22%) proteins were hypothetical proteins, seven (30%) were proteins with an unclassified function. One gene product (4.3%) is involved in central intermediary metabolism and one (4.3%) in energy metabolism. Four of the genes (17%) were pseudo genes in Tohama I. The RD-5 contained seven genes (see Additional file 4), including three (43%) genes, classified in the category transport and binding, coding for proteins involved in iron transport (Table 2). Two genes (29%) in RD-5 are pseudo genes in Tohama I (Table 2). RD-3 and RD-5 are both flanked by IS*481 *in Tohama I, on one side and both sides, respectively.

**Table 2:** Classification of Genes in RDs.

**Gene class**	**RD-3**	**RD-5**	**RD-6**	**RD-27**	**RD-10**	**RD-28**	**RD-29**	**RD-18**	RD-1*	**Tot in RD**	**Tohama I**	**% in RD**
Amino acid biosynthesis						1				1	135	4%
Biosynthesis of cofactors, prosthetic groups, and carriers			1		1					2	109	3%
Cell envelope	4	1	3		1		2			11	505	13%
Cellular Processes			1				1	1		3	169	4%
Central Intermediary metabolism	1		1		1					3	174	5%
DNA Metabolism							2			2	123	3%
Energy Metabolism	1		1		4					6	429	11%
Hypothetical proteins	5		2	1	1		2	2	2	15	260	5%
Mobile and extrachromosomal element functions					1					1	52	1%
Protein synthesis		1								1	186	5%
Regulatory funcions	3		2		1		2			8	322	8%
Transport and binding	2	3			4	1	3	1		14	327	9%
Unclassified	7	2	8		5		5	5		32	940	25%
(pseudogenes)	4 (17%)	2 (28%)	1 (5.2%)		5 (26%)	2 (100%)	3 (17.6%)	1 (11%)		18 (18%)	368	10%
Total nr of genes in RD	23	7	19	1	19	2	17	9	2	99	3731	

The number of genes belonging to COG classes is indicated for each RD, for all RDs combined (Tot in RD) and for Tohama I [26]. In the last column the % of each class present in all RDs relative to Tohama I is indicated.

The *ptxP1 *and *ptxP3 *lineages were distinguished by RD-10, a region comprising 18 genes and 22.7 kb (Table 2, and see Additional file 5). RD-10 was present in all *ptxP1 *strains analyzed. The *ptxP3 *allele is linked to two *fim3 *alleles, *fim3-1 *and *fim3-2*, and RD-10 is absent in all strains belonging to these lineages. Hierarchical clustering based on CGH data using Pearson correlation grouped the *ptxP1 *and the *ptxP3 *lineages separately (not shown). Of the 18 genes in RD-10 (see Additional file 5), four (21%) genes were classified in energy metabolism, four (21%) genes were involved in transport and binding. Further several single genes (5%) coded for a putative exported protein, a transcriptional regulator and a protein involved in iron transport, respectively (Table 2). Five of the 18 genes (26%) were pseudo genes in Tohama I. RD-10 is flanked by IS*481 *elements on both sides in the Tohama I strain.

Four genes present in RD-10 (BP1955-BP1958) were duplicated in the Tohama I genome at positions BP0579-BP0582 [26]. In agreement with this, the log ratio values for these genes in strains missing RD-10, were intermediate between those for genes which were absent or present as a single copy, respectively. In contrast, identical log ratios were observed between Tohama I and *ptxP1 *strains indicating that the genes encoded by BP0579-BP0582 are also present at positions BP1955-BP1958 in *ptxP1 *strains.

*B. pertussis *strains carrying the *ptxP3 *allele were isolated for the first time in the Netherlands in 1988. In order to analyze the homogeneity of the *ptxP3 *strains further, we extended our microarray based CGH analysis to six strains carrying the *ptxP3 *allele isolated prior to 1993 (data not shown). As was observed for strains from the period 1993 to 2004, RD-10 was missing in all 6 strains. An additional deletion of two genes (RD-1*, BP0515-BP0516) was observed in four of the early *ptxP3 *strains. The genes in BP0515 and BP0516 encode for a phage-related hypothetical protein and a hypothetical protein respectively (see Additional file 6).

The presence of RD-10 was investigated with PCR in, respectively, 20 and 35 additional *ptxP1 *and *ptxP3 *strains. This set of strains included isolates from Denmark, Finland, France, Sweden and the Netherlands. In all cases the absence of RD-10 was linked to the *ptxP3 *allele.

Interestingly, in strains missing RD-10, the PCR spanning the deletion resulted in two distinct PCR fragments (sizes approximately 1.9 and 2.9 kb). DNA sequencing showed that in the 1.9 kb PCR fragment the genes flanking RD-10, BP1946 and BP1969, were separated by one copy of IS*481*, while in the 2.9 kb PCR fragment, two copies of the IS*481 *separated these genes (data not shown). The second type was found only in strains isolated in 1993–1997.

A number of RDs were found infrequently in 11 strains analyzed with CGH and were not linked to a particular lineage as defined by MLST or MLVA; RD-6 (19 genes, 18.7 kb), RD-27 (1 gene, 0.8 kb), RD-28 (2 genes, 1.7 kb), RD-29 (17 genes, 16.9 kb) and RD-18 (9 genes, 9.4 kb) (See also Additional files 7, 8, 9, 10 and 11). Sequencing analysis showed that only part (0. 2 kb) of the RD-27 gene was deleted. The five RDs contained 48 genes of which four (8%) coded for proteins involved in transport and binding, five (10%) for cell envelope proteins and four (8%) for regulatory proteins. Seven (15%) of these 48 genes are pseudo genes (Table 2). All loci were flanked by IS*481 *sequences on at least one side in the Tohama I strain. RD-6, RD-28 and RD-29, were each found in a single strain belonging to the *ptxP1 *lineage. RD-27 was found in 6 strains of the *ptxP1 *lineage. Finally, RD-18 was found in both the *ptxP1 *lineage and the *ptxP3 *lineage in, respectively, 1 and 4 strains. The number of pseudo genes found in the RDs (18%) was higher compared to the whole genome (10%) [25].

## Discussion

In the Netherlands the upsurge of pertussis was characterized by the expansion of strains carrying a novel allele for the Ptx promoter (*ptxP3*), which completely replaced the resident *ptxP1 *strains [20,21]. It is possible that the *ptxP3 *allele has increased bacterial fitness and contributed to the upsurge of pertussis. However, the *ptxP3 *allele may also be linked to other polymorphic loci important for fitness. In this study we have characterized the *ptxP1 *and *ptxP3 *lineages using MLVA, MLST and CGH, to establish genetic relationships and to identify loci uniquely associated with *ptxP3 *strains. Further, this study highlights the microevolution of *B. pertussis *within closely related lineages.

The genetic diversity, based on MLVA frequencies, of the *B. pertussis *population showed a gradual decrease in the period 1993 to 2004 from 80% to 40%. This reflects the expansion of *ptxP3*-*fim3-2 *strains, the population of which was less diverse than the *ptxP1*-*fim3-1 *and *ptxP3*-*fim3-1 *populations (genetic diversities, respectively, 44%, 63% and 71%). The low genetic diversity of the *ptxP3*-*fim3-2 *population may reflect recent the expansion of a single clone, consistent with its recent appearance in the Dutch population.

The emergence of *fim3-2 *has also been observed in Canada and in Russia [18,19]. In view of the many surface antigens expressed by *B. pertussis*, it is remarkable that a single amino acid change in *fim3 *may affect the competitive balance between strains. The alleles for the Ptx S1 subunit and Prn code for protein variants which differ in one to seven amino acids [9,27], yet large changes in allele frequencies have been observed over the years, also suggesting that minor mutations may have a noticeable effect on strain fitness.

Consistent with studies from other groups [23,25], we observed a number for RDs comprising large deletions in *B. pertussis *isolates which were flanked by one or more IS*481 *copies. A comparison of these studies is complicated by the different designations used for the RDs. We propose a common nomenclature for the RDs observed in *B. pertussis *according to the article by Brinig *et al*, [23]. In the latter work, 26 RDs were described and we propose that novel RDs should be numbered starting from RD-27. Here RDs were numbered according to Brinig *et al *[23].

Most frequent were deletions in RD-3, RD-5 and RD-10. RD-3 and RD-5 were absent from all analyzed strains isolated in the period 1993 to 2004. RD-5 contains several genes that are predicted to be involved in iron uptake, which is essential for survival of most bacteria in the host. However, the *B. pertussis *genome contains several distinct gene clusters involved in iron uptake not all of which may be essential [26]. The absence of RD-10 was associated with *ptxP3 *strains. RD-10 is comprised of 18 genes, four of which are duplicated and found elsewhere in the Tohama I chromosome: maleate cis-trans isomerase, a probable hydrolase (a pseudo gene in Tohama I), a conserved hypothetical protein and a putative isochorismatase. CGH and PCR analyses indicated that these four genes are duplicated in all strains analyzed belonging to the *ptxP1 *lineage. The duplication of part of RD-10 may have allowed the preservation of genes important for fitness. Among the RD-10 genes lost in the *ptxP3 *lineage are those encoding for an ABC transporter system an iron uptake protein a putative transcriptional regulator and a putative exported protein. The deleted segment did not harbor any known virulence-related genes, nevertheless some of these genes may be involved in virulence. ABC transporter components can be surface associated and may be involved in virulence [28].

In the *ptxP3 *strains the region flanking the RD-10 deletion occurred in two forms. In one form the DNA regions flanking the deletion were separated by one IS*481 *copy and in the second form by two IS*481 *copies. The form with two IS*481 *copies was found mostly in strains isolated in 1993–1997 but not in strains isolated after that date. All strains isolated after 1998 were found to have the one copy of IS*481 *in this region. This suggests that the form with two copies of the IS*481 *is the precursor form, from which the second form arose after a second recombination step.

A number of RDs were deleted from one to six strains. The RD-18 deletion was observed in two strains characterized by the allele combinations *ptxP1-fim3-1 *and *ptxP3*-*fim3-2*, respectively, and these deletions therefore may reflect independent genetic events, suggesting that deleting RD-18 is beneficial for the bacterium.

As yet it is not clear if and how these deletions affect fitness. As observed by others [23-25], we found the deleted regions to be enriched for pseudo genes compared to the genome, suggesting (part of) the deleted regions were not essential. Further, the loss of (functional) genes may increase fitness by lowering metabolic costs and the number of immune targets. In several bacterial species such as *Shigella, Chlamydophila, Mycobacterium tuberculosis, Yersinia pestis *and *Salmonella enterica*, the absence of certain genes has been correlated with some beneficial effect such as an increase in pathogenicity or the onset of virulence [29].

## Conclusion

**Figure 6:**
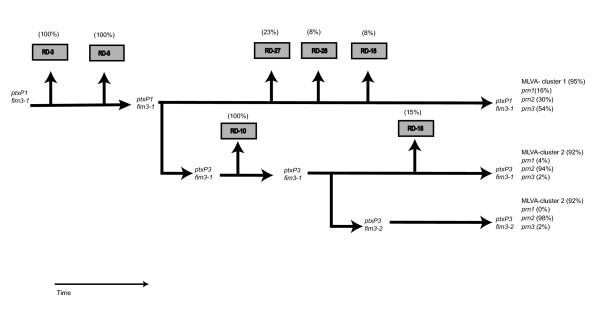
**Model for the evolution of the Bordetella pertussis ptxP1 and ptxP3 lineages**. The tree is mainly based on two point mutations found in *ptxP *and *fim3 *resulting in three lineages, *ptxP1*-*fim3-1, ptxP3*-*fim3-1 *and *ptxP3*-*fim3-2*. A single strain carrying the *ptxP1*-*fim3-4 *alleles was not included. The deleted regions are indicated in boxes and the frequency in the population is shown above the boxes. The frequencies of prn and MLVA types are given in the right part of the figure. See text for further details.

Based on the combined results of the three typing methods, we present a model for the evolution of the *ptxP1 *and *ptxP3 *lineages (Fig. 6). It was assumed that point mutations preceded the deletion of RDs. Further variation in Prn was not used to derive relationships between strains, as it is caused by reversible insertion and deletion of repeat units and hence not a useful long-term phylogenic marker [9]. Thus the tree is mainly based on two point mutations found in *ptxP *and *fim3 *resulting in three lineages, *ptxP1*-*fim3-1, ptxP3*-*fim3-1 *and *ptxP3*-*fim3-2*. The assumption that variation in Prn occurs at relative high frequency and is reversible, is reflected by the occurrence of all three detected variants in the *ptxP1*-*fim3-1 *and *ptxP3*-*fim3-1 *lineages (Fig. 6). However, the frequencies clearly differ, Prn3 and Prn2 predominating in, respectively, *ptxP1*-*fim3-1 *and *ptxP3*-*fim3-1*. Most of the strains in the *ptxP3*-*fim3-2 *lineage harbored Prn2 (98%), while Prn1 was absent (Fig. 6). Clustering based on CGH and MLVA both indicated that *ptxP1 *and *ptxP3 *strains represented distinct lineages. The *ptxP3*-*fim3-1 *and *ptxP3*-*fim3-2 *strains could not be distinguished by CGH or MLVA, possibly reflecting recent decent from a common ancestor. Since *ptxP3 *was first detected relatively recently, in 1989, we presume that this allele was derived from *ptxP1*, which predominated in the period 1950 to 1989. As *fim3-2 *was not detected before 1996, it seems likely that a mutation in a *ptxP3*-*fim3-1 *strains resulted in the *ptxP3-fim3-2 *lineage, and this was indicated in Fig. 6. We cannot exclude the alternative hypothesis, however, that the *ptxP3 *allele arose twice, in a *fim3-1 *and *fim3-2 *background. An event which occurred before the branching off of *ptxP3*, was the deletion of RD-3 and RD-5, two regions which are absent from all the Dutch strains analyzed from the period 1993 to 2004. The absence of RD-10 was characteristic for the *ptxP3 *lineage suggesting that its deletion preceded the point mutation in *ptxP3 *or that it occurred early in the history of this lineage (as assumed in Fig. 6). It is also possible that the deletion conferred a strong selective advantage resulting in clonal expansion and replacement of *ptxP3 *strains still harboring RD-10. An analysis of strains from different European countries by PCR also revealed that RD-10 was absent in *ptxP3 *strains. Conversely, RD-10 was present in all European *ptxP1 *strains studied (results not shown).

Brinig *et al*. [23], also analyzed Dutch *ptxP1 *strains with CGH and did not observe deletion of RD-10 in these strains. This study did not include any Dutch *ptxP3 *strains. Strains in which RD-10 was deleted were found in Australia, Italy and the USA [23]. Similarly, Caro *et al*. [24] and Heikkinen *et al*. [25] described the loss of RD-10 in *B. pertussis *isolates in French and Finnish strains (designated RD-4 and locus 3, respectively). In the latter paper the loss of RD-10 was found in all strains of a certain PFGE profile (SR11) that is associated with nationwide epidemics. It would be interesting to determine if in other countries RD-10 loss is linked to *ptxP3 *as shown in our study.

## Methods

### Strains

*B. pertussis *isolates were selected from the strain collection of the Dutch National Institute of Public Health. For this study we focused on *B. pertussis *isolates isolated between 1993 and 2004. The selection criteria included the location of isolation and serotype. A total of 222 strains were used for MLVA-typing [20], 133 for MLST typing [9,13], and 43 for CGH analysis (See Additional file 12). The strains selected for CGH analysis were all typed with MLVA and MLST and were representative of the most prevalent MLVA- and MLST-types from 1993 to 2004. This work does not require approval of the ethical commission.

### MLVA- typing and MLST-typing

MLVA typing was essentially performed as described previously in Schouls *et al*, 2004). For MLST, gene fragments of the pertactin gene (*prn*), the pertussis toxin promoter (*ptxP*) and the fimbriae 3 gene (*fim3*) were sequenced essentially as described previously [15,18,20]. For the MLST designation the different *fim3 *alleles were numbered as follows: *fim3A *= *fim3-1, fim3B *= fim3-2, fim3C = fim3-3 and fim3A* = *fim3-4*. For each strain, every unique sequence of the *ptxP*, *fim3 *and *prn *loci received a distinct allele number. The compositions of these loci were expressed in an allelic profile based on the allele numbers. E.g. *ptxP3*-*fim3-1-prn2*, was abbreviated to 312. The MLVA and MLST profiles were clustered with the Bionumerics software by using a categorical coefficient and a graphing method called minimum spanning tree [20].

### Culture of strains and DNA isolation

The *B. pertussis *strains used for microarray analysis in this study are listed in Additional file 12. Strains were cultured for 72 hours on Bordet-Gengou agar plates at 35°C. Subsequently, they were grown at 35°C in Verweij medium (NVI, Netherlands) with 200 μg ml^-1 ^heptakis (2,6-di-o-methyl)-β-cyclodextrine for 24 hours while being shaken at 200 rpm. Chromosomal DNA was isolated using the Promega Wizard^® ^Genomic DNA Purification Kit (Promega, Madison, USA) according to the manufacturer's instructions. The precipitated DNA was dissolved in 100 μl of EB (elution buffer, 10 mM Tris, pH 8.0) (Qiagen, Hilden, Germany).

### *B. pertussis *DNA array design and construction

70-mer oligonucleotides were selected from the complete genome sequence of *B. pertussis *strain Tohama I in collaboration with OPERON by using microarray probe design methodology as described by OPERON [30]. Each oligonucleotide was designed with an optimal specificity and sensitivity to its target gene and has an optimized Tm value. A total of 3751 unique 70-mer oligonucleotides, representing 94% of the genes of a *B. pertussis *Tohama I strain, were chosen to form the microarray. In addition, 97 oligonucleotides derived from intergenic sequences and 34 control oligonucleotides were included in the microarray. All oligonucleotides were dissolved in 50% dimethyl sulfoxide and spotted on UltraGAPS coated slides in three replicates (Corning, NY, USA) by ServiceXS (Leiden, Netherlands).

### Labeling of genomic DNA

For each CGH hybridization we labeled the DNA of a test strain and the reference strain ATCC BAA-589 Tohama I. We mixed 4 μg chromosomal DNA of *B. pertussis *with 20 μl of the 2.5 × Random Primer Mix (BioPrime DNA labeling kit; Invitrogen) in a total volume of 41 μl of water, boiled the mixture for 5 minutes, and then placed it on ice. The samples were centrifuged for 2 minutes at 13,200 rpm and were mixed with 5 μl 10× dNTP mix [2 mM dATP, dCTP and dGTP, 0.5 mM dTTP (Roche)], 2.5 μl of 1 mM Cy3 dUTP (for reference strain) or Cy5 dUTP (for the test strain) (Amersham Biosciences, UK) and 1 μl Klenow polymerase (40 U μl^-1^) (BioPrime DNA labeling kit; Invitrogen). The samples were incubated for 3 hours at 37°C. Subsequently, the test and reference samples were purified separately using CyScribe GFX Purification Kit (Amersham Biosciences, UK) and then eluted in 60 μl elution buffer. The incorporation of the Cy dyes in the labeled target sequences was measured with a NanoDrop spectrophotometer (NanoDrop Technologies). For each CGH experiment equal amounts of Cy3 dye (reference) and Cy5 dye (test) were used. The volume of the samples was decreased using a speedvac concentrator (New Brunswick Scientific, Edison, USA). The labeled test and reference samples were mixed together with 79.2 μl of hybridization solution [3.44 × SSC (Invitrogen), 0.32 % SDS (Invitrogen), 1.0 mg yeast tRNA/ml]. Before loading on the microarray the hybridization solution was heated for 3 minutes at 100°C and 5.8 μl of 10 × DIG blocking buffer was added.

### Microarray hybridization

Before hybridization of the microarrays, 600 mJ of UV energy was applied to the Corning microarrays using a UV-chamber (Bio-Rad, Richmond, CA, USA) and the microarrays were prehybridized for 45–60 minutes at 42°C in pre-warmed prehybridization solution (5× SSC, 0.1% SDS and 0.1 mgml^-1 ^BSA). The prehybridized microarrays were washed twice for 5 minutes with 0.1× SSC at room temperature. Finally the microarrays were washed with purified water for 30 seconds and were dried by blowing air with a Quick-Dry-filtered Air Gun (Matrix Technologies Corporation, Hudson, NH, USA). The labeled DNA hybridization mixture was applied to the microarray using lifterslip coverslips and hybridization was carried out in a Genemachines Hybchamber (Genomic Solutions, Ann Arbor, MI, USA) for 14–18 hours at 60°C in a water bath. The arrays were then disassembled in 2× SSC, 0.1% SDS at 60°C and washed for 5 minutes with 2× SSC, 0.1% SDS, in a hybridization oven at 60°C, followed by two washes 0.1× SSC, 0.1% SDS for 5 minutes at room temperature and four washes with 0.1× SSC at room temperature for one minute. Finally the slides were washed for 10 seconds in 0.01× SSC and then dried using a Quick-Dry-filtered Air Gun (Matrix Technologies Corporation, Hudson, NH, USA). Slides were scanned using a ScanArray 4000XL microarray scanner (Packard BioChip Technologies/Perkin Elmer) equipped with ScanArray express software.

Eighteen and 25 strains analyzed were hybridized at 42°C and 60°C, respectively. Essentially identical results were obtained, but the higher wash temperature resulted in a lower background and these results are shown in Fig. 5. All subsequent washes were performed according to the manufacturer's instruction (Corning, NY, USA).

### Image and Data analysis

The images were analyzed using Genepix Pro 5.1 software (Axon Instruments). Two-color array image data were submitted to an in-house microarray database. Raw microarray signal data (no background subtracted) were collected from the database, and after exclusion of control and blank spots the data were normalized in R [31]. The normalization employed a four-step approach consisting of: (1) log2-transformation, (2) quantile normalization of all scans, (3) calculating log2 (Cy5/Cy3) ratios per spot, and (4) taking the median from replicate spots. Visualizing the normalized log2-ratios as density plots and quantile-quantile plots showed an approximately normal distribution with an average of 0.012 and a standard deviation of 0.315. Based on this normal distribution, setting a cutoff for deletion at -1 would predict deleted genes with a False Discovery Rate (FDR, [32]) of 7.7 %; setting the cutoff at -1.5 corresponds to a FDR of 0.014%. On the basis of preliminary PCR validations, we determined a cutoff for the normalized intensity ratio of < -1.5, indicating the absence of the gene. These genes were selected for further analysis with PCR or sequence analysis. The combined normalized data were visualized with TIGR MultiExperimentViewer [33]. The logarithm of the hybridization ratio [log2 (Cy5/Cy3)] is indicated in the yellow-black-blue color scale. [log2 (Cy5/Cy3)] = -3 = Yellow, [log2 (Cy5/Cy3)] = 0 = Black, [log2 (Cy5/Cy3)] = +3 = Blue.

### PCR confirmation and sequence analysis

PCR analysis was employed in order to confirm the results predicted by the microarray hybridizations. The PCR primers were designed to target the flanking regions of each deletion so that the amplified region spanned the missing locus. The primers used in this study are listed in Table 3. The PCRs were performed under the following conditions: 20 μl total reaction volume, 10 μl Hotstart (Qiagen), 10 pmol of each primer (Eurogentec, Seraing, Belgium), 10 ng chromosomal DNA and 5% DMSO or 1.0 M Betaine (Sigma, St. Louis, USA). The amplification was carried out in a Geneamp PCR system 9700 thermocycler (Applied Biosystems, Foster City, USA) according manufacturer's recommendations. After amplification, 10 μl of each PCR product was observed via 1% agarose gel electrophoresis with SYBR-Safe (Molecular Probes, Carlsbad, USA) staining. To determine the deletion boundaries, PCR products were purified using ExoSAP-IT^® ^(USB, Cleveland, USA). Subsequently the purified PCR products were sequenced using standard Big Dye Terminator v 3.1 (Applied Biosystems, Foster City, USA). Nucleotide sequencing was performed with an Applied Biosystems 3700 DNA Analyzer. Sequence data obtained from the ABI-3700 was compared to the *B. pertussis *sequence Tohama I using the DNA-sequence analysis program KODON to determine the precise location of each deletion.

**Table 3:** Primers used in this study.

**Name**	**Description**	**Sequence (5'-3')**
BP0910-F		CGGGGTGGGGATGAGCAAT
BP0934-R		CCACGTTTTCACCCACCCAGA
BP1135-F		GGCCAGGTTCTCCTTGGCG
BP1141-R		GCGCGTGATAGTCGGCCAG
BP1156-F		GTCGAACAGGGAGACCTGGTGC
BP1178-R		GCAGTGGAGCCCCGGTTTC
BP1553-F		GGATGGCGACCGCTTTCTTG
BP1553-R		AGCATGCCGCATTTTTCATCG
BP1946ak-2F		ATCATATCCCGCGCGTCCAG
BP1969ak-2R		GCCCGAACAGCCCAGGATC
BP1948ak-1R		TAGGCCCCCATGGTGGACTG
FWBP46-69seq3	Sequence primer	CAAATGGCTGGGCCGCTTCCTGG
FWBP46-69seq4	Sequence primer	TCCCCAGCGCCGTCCAGTTCCTC
2408seq-F	Sequence primer	ATGACGGCGTCAAACCCACC
2881seq-F	Sequence primer	GCGCGGGTGACAGATGGAG
1858seq-F	Sequence primer	AAGCTGGGACGTATCCAGCGC
1554seq-R	Sequence primer	CCAGCCATTTGCGCACAGTC
2211seq-F	Sequence primer	CTTGCGTGAGTGGGCTTACGC
BP2120-F2		TGGCGGAAAGCCGCTACCT
BP2124-R2		TACGACATTCCCGGTGCCTTG
BP2820-F2		TGTCCAATTCCCTGGTGCTGG
BP2840-R		AAAGAGGCCTTGTTCCGCGAA

## Abbreviations

CGH: Comparative Genomic Hybridization; MLST: Multi-Locus Sequence Typing; MLVA: Multiple-Locus Variable number tandem repeat Analysis.

## Authors' contributions

AJK, initiated and contributed to the design of the oligonucleotides used for the microarray, setup, designed and carried out (part of) the microarray studies, participated in the MLST-, MLVA -and microarray data analysis and wrote the manuscript. TvG, carried out the MLST, MLVA and microarray experiments and commented on the manuscript. JLAP participated in the statistical analysis. HGJvdH participated in the MLST, MLVA and microarray data analysis. QH initiated and contributed to the development of the oligonucleotides used for the microarray and commented on the manuscript. DD participated in analysis of data. KH participated in the MLST analysis. MvG participated in the MLVA analysis. KvL participated in the microarray studies. FRM contributed to the conception and design of experiments and was involved in writing the manuscript. All authors read and approved the final manuscript.

## Supplementary Material

Additional file 1 Composition of MLVA types in *B. pertussis *strains Click here for file

Additional file 2 Distribution of MLVA types per year Click here for file

Additional file 3 Annotation of genes missing in circulating strains, from 1993–2004, RD-3 Click here for file

Additional file 4 Annotation of genes missing in circulating strains, from 1993–2004, RD-5 Click here for file

Additional file 5 Annotation of genes missing in circulating strains, from 1993–2004, RD-10 Click here for file

Additional file 6 Annotation of genes missing in circulating strains, from 1988–1990, RD-1* Click here for file

Additional file 7 Annotation of genes missing in circulating strains, from 1993–2004, RD-6 Click here for file

Additional file 8 Annotation of genes missing in circulating strains, from 1993–2004, RD-27 Click here for file

Additional file 9 Annotation of genes missing in circulating strains, from 1993–2004, RD-28 Click here for file

Additional file 10 Annotation of genes missing in circulating strains, from 1993–2004, RD-29 Click here for file

Additional file 11 Annotation of genes missing in circulating strains, from 1993–2004, RD-18 Click here for file

Additional file 12 *B. pertussis *strains used in this study Click here for file
