# Correction: Comparative genomic profiling of Dutch clinical *Bordetella pertussis *isolates using DNA microarrays: identification of genes absent from epidemic strains

**DOI:** 10.1186/1471-2164-11-196

**Published:** 2010-03-24

**Authors:** Audrey J King, Tamara van Gorkom, Jeroen LA Pennings, Han GJ van der Heide, Qiushui He, Dimitri Diavatopoulos, Kees Heuvelman, Marjolein van Gent, Karin van Leeuwen, Frits R Mooi

**Affiliations:** 1 Laboratory for Infectious Diseases and Screening (LIS) Centre for Infectious Disease Control, National Institute for Public Health and the Environment (RIVM), Bilthoven, The Netherlands; 2 Laboratory for Health Protection Research, National Institute for Public Health and the Environment (RIVM), Bilthoven, The Netherlands; 3 Pertussis Reference Laboratory, National Public Health Institute, Finland; 4 Department of Microbiology and Immunology, University of Melbourne, Victoria, Australia

## Correction

After the publication of this work [1] we found out that Table 1 was not filled in correctly in the original article. The total number of strains analyzed in the *ptxP1 *or the *ptxP3 *lineage was not shown between the brackets, leading to incompleteness of the data. The revised Table 1 is shown below.

**Table 1:** Presence of regions of difference in Dutch clinical isolates from 1993 to 2004

Region of difference	BP-number	No. of genes	Size		*ptxP1 *lineage	*ptxP3 *lineage
					
Locus:			(kb)			
*RD-3*	BP0910A-BP0937	23	24.9		0% (17)	0% (26)

*RD-5*	BP1135-BP1141	7	8.6		0% (17)	0% (26)

*RD-6*	BP1158-BP1176	19	18.7		94% (17)	100% (26)

*RD-27*	BP1553	1	0.8		82% (17)	100% (26)

*RD-10*	BP1948-BP1966	18	22.7		100% (27)	0% (53)

*RD-28*	BP2122-BP2123	2	1.7		94% (17)	100% (26)

*RD-29*	BP2822-BP2839	17	16.9		94% (17)	100% (26)

*RD-18*	BP3314-BP3322	9	9.4		94% (17)	92% (26)

RD-1*	BP0515-BP0516	2	1		0% (17)	13% (32)

Regions of difference (RDs) are numbered according to Brinig *et al *[23]. RD-1* is part of RD-1 as was found by Brinig *et al *[23]. The percentage of strains harbouring the region and the number of strains analyzed are indicated. Size, refers to the size of the deletion.

We regret any inconvenience that the incompleteness of Table 1 in the original article might have caused.
