# Supplementary material for: Comparative genomic profiling of Dutch clinical Bordetella pertussis isolates using DNA microarrays: Identification of genes absent from epidemic strains
Source: BMC Genomics. 2008 Jun 30;9:311. doi: 10.1186/1471-2164-9-311 (PMC2481270; doi:10.1186/1471-2164-9-311)
Supplement: Additional file 1 — Composition of MLVA types in B. pertussis strains [file 1471-2164-9-311-S1.doc]

***Additional file 1***

***Composition of MLVA types in B. pertussis strains***

|  | | No of repeats at VNTR locus: | | | | | |
| --- | --- | --- | --- | --- | --- | --- | --- |
| MLVA-type | Frequency | VNTR 1 | VNTR3a | VNTR3b | VNTR4 | VNTR5 | VNTR6 |
| 1 | 1 | 2 | 3 | 0 | 4 | 3 | 5 |
| 4 | 1 | 4 | 7 | 0 | 7 | 6 | 9 |
| 5 | 1 | 6 | 7 | 0 | 7 | 6 | 7 |
| 6 | 1 | 6 | 7 | 0 | 7 | 5 | 9 |
| 7 | 1 | 7 | 7 | 0 | 5 | 6 | 9 |
| 8 | 1 | 7 | 7 | 0 | 6 | 6 | 3 |
| 9 | 4 | 7 | 7 | 0 | 7 | 6 | 3 |
| 10 | 1 | 7 | 7 | 0 | 7 | 6 | 9 |
| 12 | 1 | 7 | 7 | 0 | 8 | 6 | 9 |
| 14 | 1 | 8 | 3 | 0 | 7 | 6 | 9 |
| 15 | 1 | 8 | 5 | 0 | 7 | 7 | 7 |
| 16 | 1 | 8 | 6 | 0 | 7 | 6 | 7 |
| 18 | 2 | 8 | 6 | 7 | 7 | 6 | 7 |
| 19 | 2 | 8 | 6 | 7 | 7 | 6 | 9 |
| 20 | 1 | 8 | 7 | 0 | 1 | 6 | 7 |
| 22 | 2 | 8 | 7 | 0 | 6 | 6 | 7 |
| 25 | 1 | 8 | 7 | 0 | 7 | 5 | 7 |
| 26 | 8 | 8 | 7 | 0 | 7 | 6 | 6 |
| 27 | 90 | 8 | 7 | 0 | 7 | 6 | 7 |
| 28 | 1 | 8 | 7 | 0 | 7 | 6 | 8 |
| 29 | 66 | 8 | 7 | 0 | 7 | 6 | 9 |
| 30 | 3 | 8 | 7 | 0 | 7 | 6 | 10 |
| 31 | 2 | 8 | 7 | 0 | 7 | 7 | 9 |
| 32 | 2 | 8 | 7 | 0 | 8 | 6 | 7 |
| 34 | 4 | 8 | 7 | 0 | 8 | 6 | 9 |
| 36 | 3 | 8 | 7 | 8 | 7 | 6 | 7 |
| 37 | 8 | 8 | 7 | 8 | 7 | 6 | 9 |
| 38 | 2 | 8 | 7 | 9 | 7 | 6 | 7 |
| 39 | 1 | 8 | 8 | 0 | 7 | 6 | 9 |
| 43 | 1 | 9 | 7 | 0 | 7 | 6 | 7 |
| 44 | 1 | 9 | 7 | 0 | 7 | 6 | 9 |
| 45 | 1 | 9 | 8 | 0 | 7 | 6 | 6 |
| ***70*** | 1 | 7 | 6 | 0 | 7 | 6 | 8 |
| ***72*** | 1 | 4 | 7 | 0 | 7 | 6 | 7 |
| ***76*** | 1 | 8 | 6 | 0 | 7 | 6 | 6 |
| ***80*** | 1 | 8 | 9 | 0 | 7 | 6 | 9 |
| ***158*** | 1 | 8 | 7 | 0 | 7 | 7 | 7 |
| ***159*** | 1 | 8 | 7 | 0 | 8 | 6 | 8 |

New types are indicated in bold and underlined.
