# Supplementary material for: Comparative genomic profiling of Dutch clinical Bordetella pertussis isolates using DNA microarrays: Identification of genes absent from epidemic strains
Source: BMC Genomics. 2008 Jun 30;9:311. doi: 10.1186/1471-2164-9-311 (PMC2481270; doi:10.1186/1471-2164-9-311)
Supplement: Additional file 2 — Distribution of MLVA types per year [file 1471-2164-9-311-S2.doc]

***Additional file 2***

***Distribution of MLVA types per year***

| Count of MLVA | MLVA |  |  |  |  |  |  |  |  |  |  |  |  |  |  |  |  |  |  |  |  |  |  |  |  |  |  |  |  |  |  |  |  |  |  |  |  |  |  |  |
| --- | --- | --- | --- | --- | --- | --- | --- | --- | --- | --- | --- | --- | --- | --- | --- | --- | --- | --- | --- | --- | --- | --- | --- | --- | --- | --- | --- | --- | --- | --- | --- | --- | --- | --- | --- | --- | --- | --- | --- | --- |
| yr | 1 | 4 | 5 | 6 | 7 | 8 | 9 | 10 | 12 | 14 | 15 | 16 | 18 | 19 | 20 | 22 | 25 | 26 | 27 | 28 | 29 | 30 | 31 | 32 | 34 | 36 | 37 | 38 | 39 | 43 | 44 | 45 | 70 | 72 | 76 | 80 | 158 | 159 | Grand Total |  |
| 1993 |  |  |  | 1 |  |  |  |  |  |  |  |  | 1 | 1 |  | 1 |  |  | 6 | 1 | 5 |  |  |  |  |  | 2 |  | 1 |  |  | 1 |  |  |  |  |  |  | 20 |  |
| 1994 |  |  |  |  |  | 1 |  | 1 |  |  |  |  |  |  |  |  |  | 2 | 4 |  | 11 | 1 | 1 |  |  |  |  |  |  |  | 1 |  |  |  |  |  |  |  | 22 |  |
| 1995 |  | 1 | 1 |  |  |  |  |  | 1 |  |  |  |  |  |  |  |  | 3 |  |  | 3 |  |  | 1 | 2 |  | 1 |  |  |  |  |  |  |  |  |  |  | 1 | 14 |  |
| 1996 | 1 |  |  |  |  |  | 2 |  |  |  |  |  |  | 1 |  |  |  | 1 | 2 |  | 14 |  |  |  | 1 |  | 1 |  |  |  |  |  |  |  |  |  | 1 |  | 24 |  |
| 1997 |  |  |  |  |  |  | 2 |  |  |  |  |  |  |  |  |  |  | 1 | 9 |  | 12 |  |  |  | 1 |  | 2 |  |  |  |  |  | 1 |  |  |  |  |  | 28 |  |
| 1998 |  |  |  |  |  |  |  |  |  |  |  |  | 1 |  |  |  |  |  | 6 |  | 9 |  |  |  |  |  | 1 |  |  |  |  |  |  |  |  |  |  |  | 17 |  |
| 1999 |  |  |  |  |  |  |  |  |  | 1 | 1 |  |  |  | 1 | 1 | 1 |  | 17 |  | 6 |  | 1 |  |  |  | 1 |  |  | 1 |  |  |  |  |  | 1 |  |  | 32 |  |
| 2000 |  |  |  |  | 1 |  |  |  |  |  |  | 1 |  |  |  |  |  |  | 15 |  | 4 |  |  | 1 |  | 2 |  | 1 |  |  |  |  |  |  |  |  |  |  | 25 |  |
| 2001 |  |  |  |  |  |  |  |  |  |  |  |  |  |  |  |  |  |  | 9 |  |  | 1 |  |  |  |  |  |  |  |  |  |  |  | 1 | 1 |  |  |  | 12 |  |
| 2002 |  |  |  |  |  |  |  |  |  |  |  |  |  |  |  |  |  |  | 8 |  | 2 |  |  |  |  |  |  |  |  |  |  |  |  |  |  |  |  |  | 10 |  |
| 2003 |  |  |  |  |  |  |  |  |  |  |  |  |  |  |  |  |  |  | 7 |  |  | 1 |  |  |  |  |  | 1 |  |  |  |  |  |  |  |  |  |  | 9 |  |
| 2004 |  |  |  |  |  |  |  |  |  |  |  |  |  |  |  |  |  | 1 | 7 |  |  |  |  |  |  | 1 |  |  |  |  |  |  |  |  |  |  |  |  | 9 |  |
| Grand Total | 1 | 1 | 1 | 1 | 1 | 1 | 4 | 1 | 1 | 1 | 1 | 1 | 2 | 2 | 1 | 2 | 1 | 8 | 90 | 1 | 66 | 3 | 2 | 2 | 4 | 3 | 8 | 2 | 1 | 1 | 1 | 1 | 1 | 1 | 1 | 1 | 1 | 1 | 222 |  |
