# Supplementary material for: Comparative genomic profiling of Dutch clinical Bordetella pertussis isolates using DNA microarrays: Identification of genes absent from epidemic strains
Source: BMC Genomics. 2008 Jun 30;9:311. doi: 10.1186/1471-2164-9-311 (PMC2481270; doi:10.1186/1471-2164-9-311)
Supplement: Additional file 3 — Annotation of genes missing in circulating strains, from 1993–2004, RD-3 [file 1471-2164-9-311-S3.doc]

***Additional file 3***

***Annotation of genes missing in circulating strains, from 1993-2004, RD-3***

| ***RD-3*** | |
| --- | --- |
| ***Gene number*** | ***Gene description*** |
| BP0910A | N-terminal region of a putative decarboxylase (pseudogene) |
| BP0911 | putative decarboxylase |
| BP0912 | LysR-family transcriptional regulator (Pseudogene) |
| BP0913 | putative exported protein |
| BP0914 | probable inner membrane compone of binding-protein-depende transport system |
| BP0915 | probable inner membrane compone of binding-protein-depende transport system |
| BP0916 | putative ATP-binding protein of a transporter (Pseudogene) |
| BP0918 | conserved hypothetical protein |
| BP0919 | putative succinate-semialdehyde dehydrogenase [NADP+] |
| BP0920 | putative exported protein |
| BP0921 | citrate utilization protein B |
| BP0922 | conserved hypothetical protein |
| BP0923 | conserved hypothetical protein |
| BP0924 | putative transcriptional regulator |
| BP0925 | putative fumarylacetoacetate-family hydrolase |
| BP0926 | conserved hypothetical protein |
| BP0927 | putative exported protein |
| BP0928 | LysR-type transcriptional regulator |
| BP0929 | putative membrane protein |
| BP0930 | putative CoA ligase |
| BP0932 | conserved hypothetical protein |
| BP0933 | conserved hypothetical protein (Pseudogene) |
| BP0934 | hypothetical protein |
