# Supplementary material for: Comparative genomic profiling of Dutch clinical Bordetella pertussis isolates using DNA microarrays: Identification of genes absent from epidemic strains
Source: BMC Genomics. 2008 Jun 30;9:311. doi: 10.1186/1471-2164-9-311 (PMC2481270; doi:10.1186/1471-2164-9-311)
Supplement: Additional file 4 — Annotation of genes missing in circulating strains, from 1993–2004, RD-5 [file 1471-2164-9-311-S4.doc]

***Additional file 4***

***Annotation of genes missing in circulating strains***, from 1993-2004, RD-5

| ***RD-5*** | |
| --- | --- |
| ***Gene number*** | ***Gene description*** |
| BP1135 | alpha-ketoglutarate-depende taurine dioxygenase (Pseudogene) |
| BP1136 | ECF-family sigma factor |
| BP1137 | putative signal transduction protein |
| BP1138 | putative ferric siderophore receptor |
| BP1139 | putative iron uptake protein |
| BP1140 | putative iron uptake protein (Pseudogene) |
| BP1141 | putative iron uptake protein |
