# Supplementary material for: Comparative genomic profiling of Dutch clinical Bordetella pertussis isolates using DNA microarrays: Identification of genes absent from epidemic strains
Source: BMC Genomics. 2008 Jun 30;9:311. doi: 10.1186/1471-2164-9-311 (PMC2481270; doi:10.1186/1471-2164-9-311)
Supplement: Additional file 5 — Annotation of genes missing in circulating strains, from 1993–2004, RD-10 [file 1471-2164-9-311-S5.doc]

***Additional file 5***

***Annotation of genes missing in circulating strains, from 1993-2004, RD-10***

| ***RD-10*** |  |
| --- | --- |
| ***Gene number*** | ***Gene description*** |
| BP1947 | transposase for IS481 element |
| BP1948 | branched-chain amino acid binding protein |
| BP1949 | putative permease compone of branched-chain amino acid transport system |
| BP1950 | putative ATP binding compone of branched-chain amino acid ABC transporter |
| BP1951 | putative ATP binding compone of ABC transporter |
| BP1952 | putative cytochrome (pseudogene) |
| BP1953 | probable oxidoreductase |
| BP1954 | putative mono-oxygenase |
| **BP1955** | **maleate cis-trans isomerase** |
| **BP1956** | **probable hydrolase (pseudogene)** |
| **BP1957** | **conserved hypothethical protein** |
| **BP1958** | **putative isochorismatase** |
| BP1959 | transposase for IS1663 |
| BP1960 | probable aldehyde dehydrogenase (pseudogene) |
| BP1961 | putative flavocytochrome |
| BP1962 | putative ferrisiderophore receptor |
| BP1963 | putative transcriptional regulator (pseudogene) |
| BP1964 | not known to exist |
| BP1965 | putative exported protein |
| BP1966 | putative sulfatase (pseudogene) |
| BP1967 | not known to exist |

This gene cluster is absent in all *ptxP3* strains. Genes indicated in bold are not absent in *ptxP3* strains because they are also present in BP0579-BP0582.
