# Supplementary material for: Comparative genomic profiling of Dutch clinical Bordetella pertussis isolates using DNA microarrays: Identification of genes absent from epidemic strains
Source: BMC Genomics. 2008 Jun 30;9:311. doi: 10.1186/1471-2164-9-311 (PMC2481270; doi:10.1186/1471-2164-9-311)
Supplement: Additional file 6 — Annotation of genes missing in circulating strains, from 1988–1990, RD-1* [file 1471-2164-9-311-S6.doc]

***Additional file 6***

***Annotation of genes missing in circulating strains, from 1988-1990, RD-1****

| ***RD-1**** |  |
| --- | --- |
| ***Gene number*** | ***Gene description*** |
| BP0515 | phage-related hypothetical protein |
| BP0516 | Hypothetical protein |
