# Supplementary material for: Comparative genomic profiling of Dutch clinical Bordetella pertussis isolates using DNA microarrays: Identification of genes absent from epidemic strains
Source: BMC Genomics. 2008 Jun 30;9:311. doi: 10.1186/1471-2164-9-311 (PMC2481270; doi:10.1186/1471-2164-9-311)
Supplement: Additional file 7 — Annotation of genes missing in circulating strains, from 1993–2004, RD-6 [file 1471-2164-9-311-S7.doc]

***Additional file 7***

***Annotation of genes missing in circulating strains, from 1993-2004, RD-6***

| ***RD-6*** | |
| --- | --- |
| ***Gene number*** | ***Gene description*** |
| BP1158 | putative dioxygenase |
| BP1159 | putative 2-pyrone-4 |
| BP1160 | putative lipoprotein |
| BP1161 | putative racemase |
| BP1162 | probable LysR-family transcriptional regulator |
| BP1163 | probable short-chain dehydrogenase |
| BP1164 | putative membrane protein |
| BP1165 | sodium/solute symporter |
| BP1165A | putative membrane protein |
| BP1166 | putative muramoyltetrapeptide carboxypeptidase |
| BP1167 | putative adolase |
| BP1168 | LysR-family transcriptional regulator |
| BP1169 | putative oxidoreductase |
| BP1170 | putative exported protein |
| BP1171 | conserved hypothetical protein |
| BP1172 | putative membrane protein (Pseudogene) |
| BP1174 | hypothetical protein |
| BP1175 | putative exported protein |
| BP1176 | putative succinylglutamate desuccinylase |
