# Supplementary material for: Comparative genomic profiling of Dutch clinical Bordetella pertussis isolates using DNA microarrays: Identification of genes absent from epidemic strains
Source: BMC Genomics. 2008 Jun 30;9:311. doi: 10.1186/1471-2164-9-311 (PMC2481270; doi:10.1186/1471-2164-9-311)
Supplement: Additional file 9 — Annotation of genes missing in circulating strains, from 1993–2004, RD-28 [file 1471-2164-9-311-S9.doc]

***Additional file 9***

***Annotation of genes missing in circulating strains, from 1993-2004, RD-28***

| ***RD-28*** | |
| --- | --- |
| ***Gene number*** | ***Gene description*** |
| BP2122 | putative membrane protein (pseudogene) |
| BP2123 | phospho-2-dehydro-3-deoxyheptonate aldolase, Phe-sensitive (pseudogene) |
