# Supplementary material for: Comparative genomic profiling of Dutch clinical Bordetella pertussis isolates using DNA microarrays: Identification of genes absent from epidemic strains
Source: BMC Genomics. 2008 Jun 30;9:311. doi: 10.1186/1471-2164-9-311 (PMC2481270; doi:10.1186/1471-2164-9-311)
Supplement: Additional file 10 — Annotation of genes missing in circulating strains, from 1993–2004, RD-29 [file 1471-2164-9-311-S10.doc]

***Additional file 10***

***Annotation of genes missing in circulating strains, from 1993-2004, RD-29***

| ***RD-29*** | |
| --- | --- |
| ***Gene number*** | ***Gene description*** |
| BP2822 | conserved hypothetical protein |
| BP2823 | conserved hypothetical protein |
| BP2824 | conserved hypothetical protein |
| BP2825 | putative G R-family transcriptional regulatory protein |
| BP2826 | putative exported protein |
| BP2827 | hypothetical protein |
| BP2828 | conserved hypothetical protein (pseudogene) |
| BP2830 | putative ABC transport protein |
| BP2831 | ABC transport protein |
| BP2832 | ABC transport protein |
| BP2833 | putative membrane protein (pseudogene) |
| BP2834 | transposase (Pseudogene) |
| BP2835 | ABC transport protein |
| BP2836 | putative DNA-binding protein |
| BP2837 | putative MerR-family transcriptional regulator |
| BP2838 | putative hydrolase |
| BP2839 | exported protein |
