# Supplementary material for: Comparative genomic profiling of Dutch clinical Bordetella pertussis isolates using DNA microarrays: Identification of genes absent from epidemic strains
Source: BMC Genomics. 2008 Jun 30;9:311. doi: 10.1186/1471-2164-9-311 (PMC2481270; doi:10.1186/1471-2164-9-311)
Supplement: Additional file 11 — Annotation of genes missing in circulating strains, from 1993–2004, RD-18 [file 1471-2164-9-311-S11.doc]

***Additional file 11***

***Annotation of genes missing in circulating strains, from 1993-2004, RD-18***

| ***RD-18*** | |
| --- | --- |
| ***Gene number*** | ***Gene description*** |
| BP3314 | putative blue copper protein |
| BP3315 | copper resistance protein |
| BP3316 | copper resistance protein |
| BP3317 | putative enoyl-CoA hydratase/isomerase |
| BP3318 | conserved hypothetical protein (Pseudogene) |
| BP3319 | putative IclR-family transcriptional regulator |
| BP3320 | conserved hypothetical protein |
| BP3321 | putative amidase |
| BP3322 | putative binding-protein-depende transport protein |
