# Supplementary material for: Comparative genomic profiling of Dutch clinical Bordetella pertussis isolates using DNA microarrays: Identification of genes absent from epidemic strains
Source: BMC Genomics. 2008 Jun 30;9:311. doi: 10.1186/1471-2164-9-311 (PMC2481270; doi:10.1186/1471-2164-9-311)
Supplement: Additional file 12 — B. pertussis strains used in this study [file 1471-2164-9-311-S12.doc]

***Additional file 12***

***B. pertussis strains used in this study***

|  |  | MLST | | |  |  |  |  |  |  |  |  |  |
| --- | --- | --- | --- | --- | --- | --- | --- | --- | --- | --- | --- | --- | --- |
| KEY | ISOL.YEAR | PtxP | Fim3 | Prn_Rn1 | MLVA | RD-3 | RD-5 | RD-6 | RD-27 | RD-10 | RD-28 | RD-29 | RD-18 |
| B0393 | 1993 | 1 |  | 2 | 29 |  |  |  |  |  |  |  |  |
| B0395 | 1993 | 1 | 1 | 3 | 29 |  |  |  |  |  |  |  |  |
| B0397 | 1993 | 1 |  | 3 | 6 |  |  |  |  |  |  |  |  |
| B0398 | 1993 | 1 |  | 2 | 27 |  |  |  |  |  |  |  |  |
| B0400 | 1993 | 1 |  | 2 | 19 |  |  |  |  |  |  |  |  |
| B0401 | 1993 | 1 |  | 1 | 45 |  |  |  |  |  |  |  |  |
| B0416 | 1993 | 1 |  | 2 | 27 |  |  |  |  |  |  |  |  |
| B0417 | 1993 | 1 |  | 2 | 39 |  |  |  |  |  |  |  |  |
| B0549 | 1993 | 1 |  | 2 | 37 |  |  |  |  |  |  |  |  |
| B0550 | 1993 | 1 |  | 3 | 29 |  |  |  |  |  |  |  |  |
| B0551 | 1993 | 1 |  | 2 | 29 |  |  |  |  |  |  |  |  |
| B0552 | 1993 | 1 |  | 2 | 27 |  |  |  |  |  |  |  |  |
| B0553 | 1993 | 1 |  | 2 | 28 |  |  |  |  |  |  |  |  |
| B0556 | 1993 | 1 |  | 2 | 37 |  |  |  |  |  |  |  |  |
| B0557 | 1993 | 1 |  | 3 | 29 |  |  |  |  |  |  |  |  |
| B0547 | 1993 | 1 | 1 | 2 |  |  |  |  |  |  |  |  |  |
| B0543 | 1993 | 1 | 1 | 3 |  |  |  |  |  |  |  |  |  |
| B0544 | 1993 | 1 | 1 | 3 |  |  |  |  |  |  |  |  |  |
| B0545 | 1993 | 1 | 1 | 3 |  |  |  |  |  |  |  |  |  |
| B0546 | 1993 | 1 | 1 | 3 |  |  |  |  |  |  |  |  |  |
| B0392 | 1993 | 1 | 1 | 3 |  |  |  |  |  |  |  |  |  |
| B0394 | 1993 | 3 | 1 | 2 | 18 | - | - | + | + | - | + | + | + |
| B0396 | 1993 | 3 |  | 2 | 27 |  |  |  |  |  |  |  |  |
| B0399 | 1993 | 3 |  | 2 | 27 |  |  |  |  |  |  |  |  |
| B0548 | 1993 | 3 | 1 | 2 | 27 | - | - | + | + | - | + | + | + |
| B0554 | 1993 | 3 | 1 | 2 | 22 | - | - | + | + | - | + | + | + |
| B0343 | 1994 | 1 | 1 | 3 | 29 |  |  |  |  |  |  |  |  |
| B0344 | 1994 | 1 | 1 | 3 | 29 |  |  |  |  |  |  |  |  |
| B0346 | 1994 | 1 | 1 | 3 | 44 |  |  |  |  |  |  |  |  |
| B0350 | 1994 | 1 | 1 | 3 | 29 |  |  |  |  |  |  |  |  |
| B0351 | 1994 | 1 | 1 | 3 | 29 |  |  |  |  |  |  |  |  |
| B0352 | 1994 | 1 | 1 | 3 | 29 |  |  |  |  |  |  |  |  |
| B0353 | 1994 | 1 | 1 | 3 | 29 |  |  |  |  |  |  |  |  |
| B0354 | 1994 | 1 | 1 | 3 | 10 |  |  |  |  |  |  |  |  |
| B0356 | 1994 | 1 |  | 3 | 29 |  |  |  |  |  |  |  |  |
| B0361 | 1994 | 1 |  | 2 | 8 |  |  |  |  |  |  |  |  |
| B0362 | 1994 | 1 |  | 3 | 27 |  |  |  |  |  |  |  |  |
| B0363 | 1994 | 1 |  | 3 | 31 |  |  |  |  |  |  |  |  |
| B0365 | 1994 | 1 |  | 3 | 27 |  |  |  |  |  |  |  |  |
| B0366 | 1994 | 1 |  | 3 | 30 |  |  |  |  |  |  |  |  |
| B0530 | 1994 | 1 | 1 | 3 | 29 | - | - | + | + | + | + | + | + |
| B0538 | 1994 | 1 |  | 3 | 29 |  |  |  |  |  |  |  |  |
| B0540 | 1994 | 1 |  | 3 | 29 |  |  |  |  |  |  |  |  |
| B0542 | 1994 | 1 | 1 | 1 | 29 | - | - | + | + | + | + | + | + |
| B0368 | 1994 | 1 |  | 1 |  |  |  |  |  | + |  |  |  |
| B0523 | 1994 | 1 | 1 | 3 |  |  |  |  |  | + |  |  |  |
| B0532 | 1994 | 1 | 1 | 1 |  |  |  |  |  |  |  |  |  |
| B0519 | 1994 | 1 | 1 | 2 |  |  |  |  |  |  |  |  |  |
| B0524 | 1994 | 1 | 1 | 2 |  |  |  |  |  |  |  |  |  |
| B0526 | 1994 | 1 | 1 | 2 |  |  |  |  |  |  |  |  |  |
| B0527 | 1994 | 1 | 1 | 2 |  |  |  |  |  |  |  |  |  |
| B0520 | 1994 | 1 | 1 | 3 |  |  |  |  |  |  |  |  |  |
| B0521 | 1994 | 1 | 1 | 3 |  |  |  |  |  |  |  |  |  |
| B0523 | 1994 | 1 | 1 | 3 |  |  |  |  |  |  |  |  |  |
| B0525 | 1994 | 1 | 1 | 3 |  |  |  |  |  |  |  |  |  |
| B0345 | 1994 | 3 | 1 | 2 | 26 | - | - | + | + | - | + | + | + |
| B0358 | 1994 | 3 |  | 2 | 27 |  |  |  |  |  |  |  |  |
| B0518 | 1994 | 3 | 1 | 2 | 26 | - | - | + | + | - | + | + | + |
| B0541 | 1994 | 3 |  | 2 | 27 |  |  |  |  | - |  |  |  |
| B0359 | 1994 | 3 |  | 2 |  |  |  |  |  | - |  |  |  |
| B0522 | 1994 | 3 | 1 | 2 |  |  |  |  |  |  |  |  |  |
| B0601 | 1995 | 1 | 1 | 2 | 37 |  |  |  |  |  |  |  |  |
| B0602 | 1995 | 1 | 1 | 1 | 34 |  |  |  |  |  |  |  |  |
| B0603 | 1995 | 1 | 1 | 1 | 34 |  |  |  |  |  |  |  |  |
| B0604 | 1995 | 1 | 1 | 3 | 12 |  |  |  |  |  |  |  |  |
| B0605 | 1995 | 1 | 1 | 3 | 29 | - | - | + | - | + | + | + | + |
| B0610 | 1995 | 1 |  | 2 | 4 |  |  |  |  |  |  |  |  |
| B0611 | 1995 | 1 |  | 3 | 29 |  |  |  |  |  |  |  |  |
| B0612 | 1995 | 1 | 1 | 3 | 29 | - | - | + | + | + | + | + | + |
| B0599 | 1995 | 1 | 1 | 3 |  |  |  |  |  | + |  |  |  |
| B0598 | 1995 | 1 | 1 | 2 |  |  |  |  |  |  |  |  |  |
| B0597 | 1995 | 1 | 1 | 3 |  |  |  |  |  |  |  |  |  |
| B0599 | 1995 | 1 | 1 | 3 |  |  |  |  |  |  |  |  |  |
| B0600 | 1995 | 3 | 1 | 2 | 32 | - | - | + | + | - | + | + | + |
| B0606 | 1995 | 3 |  | 2 | 5 |  |  |  |  |  |  |  |  |
| B0607 | 1995 | 3 | 1 | 2 | 26 | - | - | + | + | - | + | + | + |
| B0608 | 1995 | 3 |  | 2 | 26 |  |  |  |  |  |  |  |  |
| B0609 | 1995 | 3 |  | 2 | 26 |  |  |  |  | - |  |  |  |
| B0613 | 1995 | 3 | 1 | 2 | 159 | - | - | + | + | - | + | + | + |
| B0596 | 1995 | 3 | 1 | 2 |  |  |  |  |  |  |  |  |  |
| B0641 | 1996 | 1 |  | 2 | 29 |  |  |  |  |  |  |  |  |
| B0654 | 1996 | 1 | 1 | 3 | 29 | - | - | + | + | + | + | + | + |
| B0776 | 1996 | 1 | 1 | 1 | 9 |  |  |  |  |  |  |  |  |
| B0779 | 1996 | 1 |  | 2 | 29 |  |  |  |  |  |  |  |  |
| B0782 | 1996 | 1 |  | 1 | 19 |  |  |  |  |  |  |  |  |
| B0785 | 1996 | 1 |  | 3 | 29 |  |  |  |  |  |  |  |  |
| B0790 | 1996 | 1 |  | 3 | 1 |  |  |  |  |  |  |  |  |
| B0939 | 1996 | 1 |  | 3 | 29 |  |  |  |  |  |  |  |  |
| B0940 | 1996 | 1 |  | 3 | 29 |  |  |  |  |  |  |  |  |
| B0941 | 1996 | 1 |  | 2 | 29 |  |  |  |  |  |  |  |  |
| B0942 | 1996 | 1 |  | 3 | 29 |  |  |  |  |  |  |  |  |
| B0943 | 1996 | 1 |  | 3 | 29 |  |  |  |  |  |  |  |  |
| B0945 | 1996 | 1 |  | 3 | 29 |  |  |  |  |  |  |  |  |
| B0946 | 1996 | 1 |  | 2 | 37 |  |  |  |  |  |  |  |  |
| B0948 | 1996 | 1 |  | 3 | 29 |  |  |  |  |  |  |  |  |
| B0950 | 1996 | 1 |  | 2 | 29 |  |  |  |  |  |  |  |  |
| B0954 | 1996 | 1 |  | 3 | 34 |  |  |  |  |  |  |  |  |
| B0955 | 1996 | 1 |  | 3 | 29 |  |  |  |  |  |  |  |  |
| B1705 | 1996 | 1 | 1 | 2 | 27 | - | - | + | + | + | + | + | + |
| B0639 | 1996 | 1 |  | 1 |  |  |  |  |  | + |  |  |  |
| B0777 | 1996 | 1 |  |  |  |  |  |  |  | + |  |  |  |
| B0800 | 1996 | 1 |  | 2 |  |  |  |  |  | + |  |  |  |
| B0640 | 1996 | 1 | 1 | 1 |  |  |  |  |  |  |  |  |  |
| B0638 | 1996 | 1 | 1 | 3 |  |  |  |  |  |  |  |  |  |
| B0646 | 1996 | 1 | 1 | 3 |  |  |  |  |  |  |  |  |  |
| B0650 | 1996 | 1 | 1 | 3 |  |  |  |  |  |  |  |  |  |
| B0775 | 1996 | 1 | 1 | 3 |  |  |  |  |  |  |  |  |  |
| B0644 | 1996 | 3 | 1 | 2 | 158 | - | - | + | + | - | + | + | + |
| B0771 | 1996 | 3 | 1 | 3 | 27 | - | - | + | + | - | + | + | + |
| B0788 | 1996 | 3 |  | 2 | 26 |  |  |  |  |  |  |  |  |
| B0792 | 1996 | 3 | 1 | 1 | 9 | - | - | + | + | - | + | + | - |
| B0949 | 1996 | 3 | 2 | 3 | 29 | - | - | + | + | - | + | + | + |
| B0653 | 1996 | 3 |  | 2 |  |  |  |  |  | - |  |  |  |
| B0797 | 1996 | 3 |  | 2 |  |  |  |  |  | - |  |  |  |
| B1713 | 1997 | 1 | 1 | 3 | 34 | - | - | + | - | + | + | + | + |
| B1714 | 1997 | 1 | 1 | 3 | 29 | - | - | + | - | + | + | + | - |
| B1722 | 1997 | 1 | 1 | 1 | 70 | - | - | + | + | + | + | + | + |
| B1764 | 1997 | 1 | 1 | 1 | 9 |  |  |  |  |  |  |  |  |
| B1765 | 1997 | 1 |  | 2 | 37 |  |  |  |  |  |  |  |  |
| B1766 | 1997 | 1 | 1 | 3 | 29 | - | - | + | - | + | + | + | + |
| B1774 | 1997 | 1 |  | 3 | 27 |  |  |  |  |  |  |  |  |
| B1777 | 1997 | 1 | 1 | 3 | 29 |  |  |  |  |  |  |  |  |
| B1782 | 1997 | 1 |  | 2 | 29 |  |  |  |  |  |  |  |  |
| B1784 | 1997 | 1 | 1 | 2 | 29 |  |  |  |  |  |  |  |  |
| B1786 | 1997 | 1 |  | 3 | 29 |  |  |  |  |  |  |  |  |
| B1789 | 1997 | 1 |  | 3 | 29 |  |  |  |  |  |  |  |  |
| B1792 | 1997 | 1 |  | 3 | 29 |  |  |  |  |  |  |  |  |
| B1793 | 1997 | 1 |  | 3 | 29 |  |  |  |  |  |  |  |  |
| B1797 | 1997 | 1 |  | 3 | 29 |  |  |  |  |  |  |  |  |
| B1798 | 1997 | 1 |  | 2 | 37 |  |  |  |  |  |  |  |  |
| B1799 | 1997 | 1 |  | 1 | 9 |  |  |  |  |  |  |  |  |
| B1779 | 1997 | 1 | 1 | 1 |  |  |  |  |  | + |  |  |  |
| B1779 | 1997 | 1 | 1 | 1 |  |  |  |  |  |  |  |  |  |
| B1716 | 1997 | 3 | 1 | 2 | 27 | - | - | + | + | - | + | + | + |
| B1763 | 1997 | 3 |  | 2 | 27 |  |  |  |  |  |  |  |  |
| B1767 | 1997 | 3 |  | 2 | 27 |  |  |  |  |  |  |  |  |
| B1769 | 1997 | 3 |  | 2 | 29 |  |  |  |  |  |  |  |  |
| B1770 | 1997 | 3 | 2 | 2 | 27 | - | - | + | + | - | + | + | + |
| B1778 | 1997 | 3 | 1 | 1 | 29 | - | - | + | + | - | + | + | - |
| B1781 | 1997 | 3 | 1 | 2 | 26 |  |  |  |  |  |  |  |  |
| B1787 | 1997 | 3 | 1 | 2 | 27 |  |  |  |  |  |  |  |  |
| B1794 | 1997 | 3 |  | 2 | 27 |  |  |  |  |  |  |  |  |
| B1796 | 1997 | 3 |  | 2 | 27 |  |  |  |  |  |  |  |  |
| B1800 | 1997 | 3 |  | 2 | 27 |  |  |  |  | - |  |  |  |
| B1758 | 1997 | 3 |  | 2 |  |  |  |  |  | - |  |  |  |
| B1773 | 1997 | 3 | 1 | 2 |  |  |  |  |  |  |  |  |  |
| B2005 | 1997 | 1 |  | 2 |  |  |  |  |  |  |  |  |  |
| B1409 | 1998 | 1 | 1 | 2 | 29 |  |  |  |  |  |  |  |  |
| B1807 | 1998 | 1 | 1 | 2 | 29 |  |  |  |  |  |  |  |  |
| B1945 | 1998 | 1 | 1 | 3 | 29 | - | - | + | - | + | + | + | + |
| B1946 | 1998 | 1 |  | 3 | 29 |  |  |  |  |  |  |  |  |
| B1947 | 1998 | 1 |  | 2 | 29 |  |  |  |  |  |  |  |  |
| B1953 | 1998 | 1 |  | 2 | 29 |  |  |  |  |  |  |  |  |
| B1954 | 1998 | 1 |  | 3 | 29 |  |  |  |  |  |  |  |  |
| B1961 | 1998 | 1 |  | 3 | 29 |  |  |  |  |  |  |  |  |
| B1962 | 1998 | 1 |  | 2 | 27 |  |  |  |  |  |  |  |  |
| B1407 | 1998 | 3 | 1 | 2 | 27 |  |  |  |  |  |  |  |  |
| B1408 | 1998 | 3 | 2 | 2 | 18 | - | - | + | + | - | + | + | + |
| B1410 | 1998 | 3 | 1 | 2 | 27 |  |  |  |  |  |  |  |  |
| B1710 | 1998 | 3 |  | 2 | 27 |  |  |  |  |  |  |  |  |
| B1810 | 1998 | 3 |  | 2 | 29 |  |  |  |  |  |  |  |  |
| B1951 | 1998 | 3 |  | 2 | 27 |  |  |  |  |  |  |  |  |
| B1960 | 1998 | 3 |  | 2 | 27 |  |  |  |  |  |  |  |  |
| B1963 | 1998 | 3 |  | 2 | 37 |  |  |  |  |  |  |  |  |
| B1805 | 1998 | 3 | 1 | 2 |  |  |  |  |  | - |  |  |  |
| B1949 | 1998 | 3 |  | 1 |  |  |  |  |  | - |  |  |  |
| B1957 | 1998 | 3 |  | 2 |  |  |  |  |  | - |  |  |  |
| B1805 | 1998 | 3 | 1 | 2 |  |  |  |  |  |  |  |  |  |
| B1411 | 1998 | 3 | 2 | 2 |  |  |  |  |  |  |  |  |  |
| B1695 | 1999 | 1 |  | 2 | 27 |  |  |  |  |  |  |  |  |
| B1819 | 1999 | 1 | 1 | 2 | 29 |  |  |  |  |  |  |  |  |
| B1820 | 1999 | 1 | 1 | 3 | 14 |  |  |  |  |  |  |  |  |
| B1825 | 1999 | 1 |  | 2 | 29 |  |  |  |  |  |  |  |  |
| B1826 | 1999 | 1 | 1 | 2 | 80 |  |  |  |  |  |  |  |  |
| B1833 | 1999 | 1 |  | 1 | 29 |  |  |  |  |  |  |  |  |
| B1834 | 1999 | 1 | 1 | 2 | 29 | - | - | + | + | + | + | + | + |
| B1837 | 1999 | 1 |  | 2 | 31 |  |  |  |  |  |  |  |  |
| B1841 | 1999 | 1 | 1 | 3 | 37 |  |  |  |  |  |  |  |  |
| B1843 | 1999 | 1 | 1 | 2 | 29 | - | - | + | + | + | + | + | + |
| B1889 | 1999 | 1 |  | 2 | 29 |  |  |  |  |  |  |  |  |
| B1690 | 1999 | 3 |  | 2 | 27 |  |  |  |  |  |  |  |  |
| B1692 | 1999 | 3 |  | 2 | 27 |  |  |  |  |  |  |  |  |
| B1693 | 1999 | 3 |  | 2 | 15 |  |  |  |  |  |  |  |  |
| B1694 | 1999 | 3 |  | 3 | 27 |  |  |  |  |  |  |  |  |
| B1700 | 1999 | 3 |  | 2 | 27 |  |  |  |  | - |  |  |  |
| B1706 | 1999 | 3 | 1 | 2 | 27 | - | - | + | + | - | + | + | + |
| B1817 | 1999 | 3 |  | 2 | 20 |  |  |  |  |  |  |  |  |
| B1818 | 1999 | 3 |  | 2 | 27 |  |  |  |  |  |  |  |  |
| B1828 | 1999 | 3 | 1 | 2 | 27 | - | - | + | + | - | + | + | - |
| B1829 | 1999 | 3 |  | 2 | 43 |  |  |  |  |  |  |  |  |
| B1831 | 1999 | 3 | 2 | 2 | 27 | - | - | + | + | - | + | + | + |
| B1835 | 1999 | 3 |  | 2 | 27 |  |  |  |  |  |  |  |  |
| B1836 | 1999 | 3 | 1 | 2 | 27 |  |  |  |  |  |  |  |  |
| B1838 | 1999 | 3 | 2 | 2 | 22 |  |  |  |  |  |  |  |  |
| B1840 | 1999 | 3 | 1 | 2 | 25 |  |  |  |  |  |  |  |  |
| B1842 | 1999 | 3 |  | 2 | 27 |  |  |  |  |  |  |  |  |
| B1844 | 1999 | 3 |  | 2 | 27 |  |  |  |  | - |  |  |  |
| B1845 | 1999 | 3 |  | 2 | 27 |  |  |  |  |  |  |  |  |
| B1846 | 1999 | 3 |  | 2 | 27 |  |  |  |  |  |  |  |  |
| B1883 | 1999 | 3 |  | 2 | 27 |  |  |  |  |  |  |  |  |
| B1890 | 1999 | 3 |  | 2 | 27 |  |  |  |  | - |  |  |  |
| B1868 | 2000 | 1 | 1 | 2 | 29 | - | - | + | - | + | - | + | + |
| B1875 | 2000 | 1 |  | 3 | 7 |  |  |  |  |  |  |  |  |
| B1878 | 2000 | 1 | 1 | 2 | 29 | - | - | + | + | + | + | + | + |
| B1920 | 2000 | 1 | 1 | 2 | 29 |  |  |  |  |  |  |  |  |
| B1861 | 2000 | 3 |  | 2 | 27 |  |  |  |  |  |  |  |  |
| B1863 | 2000 | 3 | 1 | 2 | 29 |  |  |  |  |  |  |  |  |
| B1865 | 2000 | 3 | 2 | 2 | 27 |  |  |  |  |  |  |  |  |
| B1873 | 2000 | 3 | 1 | 2 | 27 | - | - | + | + | - | + | + | - |
| B1874 | 2000 | 3 |  | 2 | 36 |  |  |  |  |  |  |  |  |
| B1877 | 2000 | 3 |  | 2 | 36 |  |  |  |  |  |  |  |  |
| B1894 | 2000 | 3 |  | 2 | 27 |  |  |  |  |  |  |  |  |
| B1895 | 2000 | 3 |  | 2 | 16 |  |  |  |  |  |  |  |  |
| B1900 | 2000 | 3 | 2 | 2 | 27 | - | - | + | + | - | + | + | + |
| B1915 | 2000 | 3 |  | 2 | 27 |  |  |  |  |  |  |  |  |
| B1916 | 2000 | 3 | 2 | 2 | 38 |  |  |  |  |  |  |  |  |
| B1917 | 2000 | 3 | 2 | 2 | 27 |  |  |  |  | - |  |  |  |
| B1918 | 2000 | 3 | 2 | 2 | 27 |  |  |  |  |  |  |  |  |
| B1919 | 2000 | 3 |  | 2 | 27 |  |  |  |  |  |  |  |  |
| B1921 | 2000 | 3 |  | 2 | 27 |  |  |  |  |  |  |  |  |
| B1924 | 2000 | 3 |  | 2 | 27 |  |  |  |  |  |  |  |  |
| B1926 | 2000 | 3 |  | 2 | 27 |  |  |  |  |  |  |  |  |
| B1927 | 2000 | 3 |  | 2 | 27 |  |  |  |  |  |  |  |  |
| B1928 | 2000 | 3 |  | 2 | 27 |  |  |  |  |  |  |  |  |
| B1929 | 2000 | 3 |  | 2 | 32 |  |  |  |  |  |  |  |  |
| B1930 | 2000 | 3 |  | 2 | 27 |  |  |  |  |  |  |  |  |
| B1862 | 2000 | 3 | 2 | 2 |  |  |  |  |  | - |  |  |  |
| B1866 | 2000 | 3 | 1 | 2 |  |  |  |  |  |  |  |  |  |
| B1867 | 2000 | 3 | 1 | 2 |  |  |  |  |  |  |  |  |  |
| B1862 | 2000 | 3 | 2 | 2 |  |  |  |  |  |  |  |  |  |
| B1870 | 2000 | 3 | 2 | 2 |  |  |  |  |  |  |  |  |  |
| B2043 | 2001 | 1 | 1 | 2 | 30 | - | - | + | + | + | + | + | + |
| B2046 | 2001 | 1 |  | 1 |  |  |  |  |  | + |  |  |  |
| B2052 | 2001 | 1 |  | 2 |  |  |  |  |  | + |  |  |  |
| B2026 | 2001 | 3 | 1 | 2 | 27 |  |  |  |  | - |  |  |  |
| B2028 | 2001 | 3 | 2 | 2 | 27 |  |  |  |  |  |  |  |  |
| B2033 | 2001 | 3 | 2 | 2 | 27 |  |  |  |  |  |  |  |  |
| B2034 | 2001 | 3 | 1 | 2 | 72 |  |  |  |  |  |  |  |  |
| B2035 | 2001 | 3 | 2 | 2 | 27 | - | - | + | + | - | + | + | + |
| B2036 | 2001 | 3 |  | 2 | 27 |  |  |  |  |  |  |  |  |
| B2037 | 2001 | 3 |  | 2 | 27 |  |  |  |  |  |  |  |  |
| B2038 | 2001 | 3 |  | 2 | 27 |  |  |  |  |  |  |  |  |
| B2039 | 2001 | 3 | 1 | 2 | 27 | - | - | + | + | - | + | + | + |
| B2040 | 2001 | 3 |  | 2 | 76 |  |  |  |  |  |  |  |  |
| B2042 | 2001 | 3 |  | 2 | 27 |  |  |  |  |  |  |  |  |
| B2050 | 2001 | 3 |  | 2 |  |  |  |  |  | - |  |  |  |
| B2031 | 2001 | 3 | 1 | 2 |  |  |  |  |  |  |  |  |  |
| B2032 | 2001 | 3 | 1 | 2 |  |  |  |  |  |  |  |  |  |
| B2027 | 2001 | 3 | 2 | 2 |  |  |  |  |  |  |  |  |  |
| B2029 | 2001 | 3 | 2 | 2 |  |  |  |  |  |  |  |  |  |
| B2030 | 2001 | 3 | 2 | 2 |  |  |  |  |  |  |  |  |  |
| B2383 | 2002 | 1 |  | 3 | 29 |  |  |  |  |  |  |  |  |
| B2414 | 2002 | 1 | 4 | 1 | 29 | - | - | - | + | + | + | - | + |
| B2407 | 2002 | 1 |  | 1 |  |  |  |  |  | + |  |  |  |
| B2390 | 2002 | 3 | 2 | 2 | 27 |  |  |  |  |  |  |  |  |
| B2396 | 2002 | 3 |  | 2 | 27 |  |  |  |  |  |  |  |  |
| B2402 | 2002 | 3 |  | 2 | 27 |  |  |  |  |  |  |  |  |
| B2409 | 2002 | 3 | 2 | 2 | 27 | - | - | + | + | - | + | + | + |
| B2410 | 2002 | 3 |  | 2 | 27 |  |  |  |  |  |  |  |  |
| B2417 | 2002 | 3 |  | 2 | 27 |  |  |  |  |  |  |  |  |
| B2570 | 2002 | 3 |  | 2 | 27 |  |  |  |  |  |  |  |  |
| B2385 | 2002 | 3 | 2 | 2 |  |  |  |  |  | - |  |  |  |
| B2400 | 2002 | 3 |  | 2 |  |  |  |  |  | - |  |  |  |
| B2384 | 2002 | 3 | 1 | 2 |  |  |  |  |  |  |  |  |  |
| B2385 | 2002 | 3 | 2 | 2 |  |  |  |  |  |  |  |  |  |
| B2386 | 2002 | 3 | 2 | 2 |  |  |  |  |  |  |  |  |  |
| B2387 | 2002 | 3 | 2 | 2 |  |  |  |  |  |  |  |  |  |
| B2388 | 2002 | 3 | 2 | 2 |  |  |  |  |  |  |  |  |  |
| B2389 | 2002 | 3 | 2 | 2 |  |  |  |  |  |  |  |  |  |
| B2391 | 2002 | 3 | 2 | 2 |  |  |  |  |  |  |  |  |  |
| B2392 | 2002 | 3 | 2 | 2 |  |  |  |  |  |  |  |  |  |
| B2393 | 2002 | 3 | 2 | 2 |  |  |  |  |  |  |  |  |  |
| B2424 | 2002 |  |  |  | 27 |  |  |  |  |  |  |  |  |
| B2575 | 2003 | 1 | 1 | 2 | 30 |  |  |  |  |  |  |  |  |
| B2572 | 2003 | 3 | 1 | 2 | 27 |  |  |  |  |  |  |  |  |
| B2573 | 2003 | 3 | 2 | 2 | 27 |  |  |  |  | - |  |  |  |
| B2579 | 2003 | 3 | 2 | 2 | 27 |  |  |  |  |  |  |  |  |
| B2582 | 2003 | 3 | 2 | 2 | 38 |  |  |  |  |  |  |  |  |
| B2583 | 2003 | 3 | 2 | 2 | 27 | - | - | + | + | - | + | + | + |
| B2584 | 2003 | 3 | 1 | 2 | 27 |  |  |  |  |  |  |  |  |
| B2591 | 2003 | 3 |  | 2 | 27 |  |  |  |  | - |  |  |  |
| B2593 | 2003 | 3 |  | 2 | 27 |  |  |  |  |  |  |  |  |
| B2578 | 2003 | 3 | 1 | 2 |  |  |  |  |  |  |  |  |  |
| B2574 | 2003 | 3 | 2 | 2 |  |  |  |  |  |  |  |  |  |
| B2580 | 2003 | 3 | 2 | 2 |  |  |  |  |  |  |  |  |  |
| B2594 | 2004 | 3 |  | 2 | 27 |  |  |  |  |  |  |  |  |
| B2718 | 2004 | 3 | 2 | 2 | 27 |  |  |  |  |  |  |  |  |
| B2724 | 2004 | 3 | 1 | 2 | 27 |  |  |  |  |  |  |  |  |
| B2740 | 2004 | 3 |  | 2 | 26 |  |  |  |  |  |  |  |  |
| B2747 | 2004 | 3 |  | 2 | 36 |  |  |  |  |  |  |  |  |
| B2752 | 2004 | 3 |  | 2 | 27 |  |  |  |  |  |  |  |  |
| B2755 | 2004 | 3 | 1 | 2 | 27 | - | - | + | + | - | + | + | + |
| B2764 | 2004 | 3 |  | 2 | 27 |  |  |  |  |  |  |  |  |
| B2771 | 2004 | 3 |  | 2 | 27 |  |  |  |  |  |  |  |  |
| B2716 | 2004 | 3 | 1 | 2 |  |  |  |  |  | - |  |  |  |
| B2749 | 2004 | 3 |  | 2 |  |  |  |  |  | - |  |  |  |
| B2716 | 2004 | 3 | 1 | 2 |  |  |  |  |  |  |  |  |  |
| B2717 | 2004 | 3 | 1 | 2 |  |  |  |  |  |  |  |  |  |
| B2719 | 2004 | 3 | 1 | 2 |  |  |  |  |  |  |  |  |  |
| B2720 | 2004 | 3 | 1 | 2 |  |  |  |  |  |  |  |  |  |
| B2715 | 2004 | 3 | 2 | 2 |  |  |  |  |  |  |  |  |  |
| B2721 | 2004 | 3 | 2 | 2 |  |  |  |  |  |  |  |  |  |
| B2723 | 2004 | 3 | 2 | 2 |  |  |  |  |  |  |  |  |  |
| B2726 | 2004 | 3 | 2 | 2 |  |  |  |  |  |  |  |  |  |
| B2776 | 2005 | 3 | 1 | 2 |  |  |  |  |  | - |  |  |  |
| B2884 | 2005 | 3 | 1 | 2 |  |  |  |  |  | - |  |  |  |
| B2888 | 2005 | 3 | 1 | 2 |  |  |  |  |  | - |  |  |  |
| B2905 | 2006 | 3 | 1 | 2 |  |  |  |  |  | - |  |  |  |

All strains in this table are Dutch clinical isolates, key number, isolation year, sequence types for ptxP, Fim3 and Prn_Rn1, MLVA type and the RDs are indicated in the headings. The presence or absence of RDs were determined by CGH- and PCR- analysis, + indicates the presence of the genes in the RD and – indicates the absence of the genes in the RD in that particular strain. Sequence types for ptxP, Fim3 and Prn_Rn1 combined in this order result in MLST typing profiles. An empty cell means that these strains were not typed.
